# Volatile Composition in Two Pummelo Cultivars (*Citrus grandis* L. Osbeck) from Different Cultivation Regions in China

**DOI:** 10.3390/molecules22050716

**Published:** 2017-04-29

**Authors:** Mingxia Zhang, Linbo Li, Zhongwei Wu, Yanjie Wang, Yiming Zang, Guojie Liu

**Affiliations:** 1 School of Life Science and Technology, Henan Institute of Science and Technology, Xinxiang 453003, Henan, China; Lilinbo@126.com (L.L.); wuzhongwei0417@sina.com (Z.W.); mailyanjie@163.com (Y.W.); 2 College of Agriculture and Biotechnology, China Agriculture University, Yuanmingyuan Xilu, Beijing 10093, China; aspringswallow@aliyun.com (Y.Z.); lgj@cau.edu.cn (G.L.)

**Keywords:** pummelo, volatile compounds, different cultivation regions, principal component analysis, cluster analysis

## Abstract

This study investigated the composition of volatile compounds in two pummelo cultivars, including ‘Shatian’ and ‘Guanxi’, cultivated in different regions of China with the aim of studying the effect of cultivar and cultivation condition on biosynthesis of volatile compounds in pummelo. Volatile compounds were extracted from pummelo juice using head-space microextraction and then analyzed using gas chromatography coupled with mass spectrometry. Results showed that a total of 49 volatile compounds was detected in the study, including 11 aldehydes, 7 alcohols, 3 ketones, 7 esters, 19 terpenes and 2 other volatiles. The ‘Guanxi’ pummelo cultivar possessed a more complex composition of volatile compounds compared with the ‘Shatian’ cultivar. Meanwhile, the volatile compounds appeared to exhibit a higher concentration in the ‘Guanxi’ cultivar samples than the ‘Shatian’ cultivar. Cluster analysis revealed that the ‘Guanxi’ cultivar samples from the different regions were grouped together, whereas the ‘Shatian’ cultivar samples were assembled. Principal component analysis showed that an obvious separation was observed between the ‘Guanxi’ and ‘Shatian’ cultivar. However, the ‘Shatian-SC15’ was significantly separated from the other ‘Shatian’ cultivar samples. These indicated that cultivar genotype was the primary factor that determined the volatile profile of the pummelo cultivar. Cultivation region might affect the biosynthesis of volatile compounds, resulting in the differentiation of the volatile composition in each pummelo cultivar.

## 1. Introduction

Volatile compounds are important secondary metabolites biosynthesized in fruits. These secondary metabolites play important roles in determination of the overall aroma of fruits [1,2,3]. Basically, volatile compounds in fruits are comprised of free and bound forms. Free volatile compounds are initially accumulated in fruits under a series of enzymatic reactions and further conjugated with the sugar moiety to yield bound volatiles [4]. Although the sugar moiety in bound volatile compounds enhances the accumulation of volatile compounds in fruits due to their improved solubility, these glycosidically-conjugated volatiles cannot directly contribute to the overall aroma of fruits since moiety conjugation results in these metabolites with odorless properties [4,5]. Therefore, it is free volatile compounds that play the essential roles in contributing their flavor notes to the fruit aroma [4]. During the fruit fermentation and storage period, enzymatic and/or chemical hydrolysis occur for bound volatile compounds, leading to the cleavage of the sugar moiety to release their volatile aglycones. These released volatile aglycones can further incorporate their aroma characteristics into fruit products [4,6]. However, the overall aroma of fruit juice, especially fresh fruit juice, is basically determined by free volatile composition [7,8,9].

Pummelo (*Citrus grandis* L. Osbeck) is a natural and non-hybrid citrus fruit that is mainly cultivated in South and Southeast Asia [10]. Pummelo has been considered the principal ancestor of grapefruits. It appears as a larger grapefruit, and its juice tastes sweeter and less bitter than grapefruit juice [11]. It has been accepted that pummelo was introduced into China around 100 B.C., and pummelo has been widely cultivated in southern China, such as Guangxi, Guangdong, Fujian and Sichuan [10]. The major volatile compounds in pummelo juice include esters, terpenoids, alcohols, aldehydes and ketones [6,11]. It has been confirmed that fruit genotype essentially determines the secondary metabolite patterns in fruits, whereas cultivation conditions also play an important role in regulating the accumulation level of secondary metabolites [12,13,14,15]. Our previous study has confirmed that the phenolic compound profile in flavedo and juice of the same pummelo cultivar exhibited regional differences [16]. In the present study, we further attempted to investigate if the genotype and cultivation conditions could also alter the composition of volatile compounds in pummelo. Therefore, we collected two pummelo cultivars grown in different regions of China and further extracted their free volatile compounds using solid phase micro-extraction (SPME). The profile of the pummelo cultivars was further analyzed and compared using gas chromatography coupled with mass spectrometry (GC-MS). The volatile profile similarity of the cultivars cultivated in different regions of China was tested using multivariate analyses, including cluster analysis and principal component analysis. The objective of this study was to elucidate the effect of cultivar genotype and cultivation condition on the volatile composition of pummelo fruits, which could provide useful information on pummelo cultivation management and optimization and bring knowledge to pummelo cultivar breeding development.

## 2. Results and Discussion

### 2.1. Physicochemical Index

Table 1 shows the fruit weight, total soluble solids, total sugar and total acid of these two pummelo cultivar samples harvested from different regions of China. The ‘Guanxi’ pummelo cultivar fruits exhibited higher fruit weight compared to the ‘Shatian’ cultivar samples. Additionally, the ‘Shatian’ cultivar pummelo samples harvested from the different regions showed similar fruit weight. The similar fruit weight was also observed in the different regions for harvested ‘Guanxi’ cultivar samples. In the meantime, the fruit weight of the ‘Shatian-GX15’ sample was similar to that of the ‘Shatian-GX5’ sample. A similar observation was also found in the ‘Guanxi-FJ15’ and ‘Guanxi-FJ5’ cultivar samples.

The ‘Guanxi’ cultivar samples from the different regions showed similar total soluble solids, whereas a difference in the total soluble solids was found in the ‘Shatian’ pummelo samples collected from different regions. For example, the ‘Shatian-GD15’ sample exhibited the highest content of the total soluble solids, whereas the lowest total soluble solids content was found in the ‘Shatian-SC15’ sample. No significant differences were found in the ‘Shatian’ cultivars harvested in the different ages of trees from the Guangxi region. A similar observation was also found in the ‘Guanxi-FJ15’ and ‘Guanxi-FJ5’ fruits. Regarding total sugar content in these cultivar samples, the ‘Shatian’ and ‘Guanxi’ cultivar fruits possessed a total sugar content ranging from 91.3 to 111.7 g/L and 98.6 to 112.5 g/L, respectively. Meanwhile, the ‘Shatian’ cultivar samples showed a lower total acidity level than the ‘Guanxi’ cultivar samples. Regional cultivation appeared to affect the accumulation of sugar and acids in the same pummelo cultivar. For example, ‘Shatian-GD15’ and ‘Shatian-GX15’ showed higher sugar content than the ‘Shatian-SC15’ sample, whereas the ‘Guanxi-GD15’ sample had less total acid content than the ‘Guanxi-FJ15’ and ‘Guanxi-SC15’ samples. It has been reported that the sugar-to-acid ratio significantly affects the taste attributes of fruits, and a higher sugar-to-acid ratio indicates a better taste [11]. In the present study, the ‘Shatian’ cultivar samples showed a much higher sugar-to-acid ratio than the ‘Guanxi’ pummelo fruits, indicating that cultivar genotype might impact the accumulation of sugar and acid in different genotypes of pummelo fruits [11,17]. More importantly, the sugar-to-acid ratios in the same cultivar harvested from the different regions were also significantly different, which indicated that regional condition also played an important role in regulating the biosynthesis of primary nutrients during fruit development stages [18,19]. It should also be noted that tree age also exerted an effect on the content of the sugar-to-acid ratio in each cultivar, and a higher ratio appeared to be found in the fruits from the younger trees.

### 2.2. Volatile Composition

Secondary metabolites, including volatile compounds, are generally biosynthesized from primary nutrients in plant as a response to fruit development and environmental changes [5,14]. Secondary metabolites can play important roles in affecting the sensory attributes and nutritional quality of fruits and provide fruits with multiple bioactive properties [14,16]. Regarding their chemical nature, volatile compounds can be divided into aldehydes, alcohols, ketones, esters, terpenes and other volatiles. In the present study, a total of 49 volatiles was detected, including 11 aldehydes, 7 alcohols, 3 ketones, 7 esters, 19 terpenes and 2 other volatiles (Table 2). Our results were consistent with the previous studies [4,6,11].

#### 2.2.1. Aldehydes

Aldehydes are one of the most important classes of volatile compounds that play important roles in providing fruits with the featured flavors [15]. Biologically, aldehydes in fruits are basically formed from the α-oxidation of fatty acids [20,21]. For example, it has been reported that linolenic acid can be converted into hexanal, (*Z*)-3-hexenal and (*E*)-2-hexenal in fruits under multiple enzymatic reactions [21].

Regarding the aldehyde composition in these two pummelo samples, the ‘Guanxi’ pummelo cultivar possessed a more complicated aldehyde composition than the ‘Shatian’ cultivars. For example, the ‘Guanxi’ pummelo samples were found to contain 11 aldehydes, except for heptanal not present in the ‘Guanxi-GD15’ or ‘Guanxi-SC15’ sample and 2-hexenal not in the ‘Guanxi-FJ5’ sample (Table 2). However, the ‘Shatian’ cultivars only contained two aldehydes, except for ‘Shatian-SC15’. These indicated that the cultivar genotype might exert a primary role in the determination of aldehyde composition in different pummelo cultivars. More importantly, the region effect significantly resulted in the composition differences on aldehydes in the ‘Shatian’ cultivar samples, whereas individual aldehyde content was significantly altered in the ‘Guanxi’ cultivars harvested from the different regions. For example, hexanal was the only aldehyde that was found in all of the ‘Shatian’ cultivar samples (Table 2). Additionally, a higher concentration of hexanal was observed in the ‘Shatian-GD15’ and ‘Shantian-SC15’ samples compared with ‘Shantian’ samples from the Guangxi region. The ‘Shatian-SC15’ sample also contained a moderate content of *E*-2-pentenal, whereas this volatile was not found in the other ‘Shatian’ samples. Similarly, *Z*-2-heptanal was only present in the ‘Shatian-GD15’ and ‘Shatian-SC15’ samples, and a significant difference in its content was observed between these two ‘Shatian’ fruits. Only the ‘Shatian-SC15’ and ‘Shatian-GX15’ fruits possessed nonanal with a similar concentration. *E*-2-octenal was only found in the ‘Shatian-15’ and ‘Shatian-GX5’ samples, and its concentration was significantly different in these fruit samples. It was also observed that the ‘Shatian-SC15’ pummelo fruit contained *E*,*E*-2,4-hexadienal and *E*,*E*-2,4-heptadienal, although their concentration was not high.

In the ‘Guanxi’ cultivar samples, hexanal appeared to be the major aldehyde, and its concentration was significantly different among the different region harvested samples (Table 2). For example, the ‘Guanxi-GD15’ pummelo sample had a hexanal concentration about two-times higher than that in the ‘Guanxi-SC15’ and ‘Guanxi-FJ15’. No significant differences in this aldehyde content was observed in the ‘Guanxi-FJ15’ and ‘Guanxi-FJ5’ samples. Additionally, *E*-2-pentenal and *Z*-2-heptenal were also present in the ‘Guanxi’ cultivar at a moderate level. Similar to hexanal, the ‘Guanxi-GD15’ sample exhibited a higher level (about two-times higher) on *E*-2-pentenal and *Z*-2-heptenal than the ‘Guanxi-SC15’ and ‘Guanxi-FJ15’ sample. Besides, the ‘Guanxi-FJ5’ sample contained a higher concentration of *E*-2-pentenal than the ‘Guanxi-FJ15’ sample, whereas these two Fujian regions samples from different ages of trees contained similar content of *Z*-2-heptenal. In addition, *E*-2-octenal, *E*,*E*-2,4-hexadienal and nonanal existed in the ‘Guanxi’ cultivar at a moderate level (Table 2). A similar content of *E*-2-octenal was found in the ‘Guanxi-GD15’, ‘Guanxi-SC15’ and ‘Guanxi-FJ15’ samples, whereas ‘Guanxi-FJ15’ and ‘Guanxi-FJ5’ did not show aby difference in the *E*-2-octenal level. A similar observation was also found in the level of nonanal among these samples. However, the ‘Guanxi-GD15’ sample contained much higher content (about two-times higher) on *E*,*E*-2,4-hexadienal than the ‘Guanxi-SC15’ and ‘Guanxi-FJ15’. Both of the ‘Guanxi-FJ15’ and ‘Guanxi-FJ5’ samples had a similar content of *E*,*E*-2,4-hexadienal. It should be made known that heptanal was only found in the ‘Guanxi’ sample harvested in the Fujian region, and the ‘Guanxi-FJ5’ sample showed a significantly higher content (about 10-times higher) of heptanal than the ‘Guanxi-FJ15’ sample (Table 2). The highest content of 2-hexenal was found in ‘Guanxi-FJ15’, followed by ‘Guxanxi-GD15’ and then ‘Guanxi-SC15’. ‘Guanxi-FJ5’ did not contain this aldehyde. Benzaldehyde and *E*-2-nonenal were found in all of the ‘Guanxi’ cultivar samples, although their level was relatively low compared to the other aldehydes (Table 2). No content differences on benzaldehyde or *E*-2-nonenal were observed among the samples harvested from the different regions.

#### 2.2.2. Alcohols

Alcohols have been reported to be formed from amino acids in fruits [22]. Amino acids experience transamination, decarboxylation and reduction/oxidation to finally yield alcohols in fruits under the activity of multiple enzymes [22]. Nonetheless, alcohols have been detected in pummelo fruits at a low level, indicating that these volatile compounds might have a limited effect on the contribution of their flavor notes to the overall aroma of pummelo fruits [2].

Regarding the composition of alcohols in the ‘Shatian’ cultivar samples, ‘Shatian-GD15’ and ‘Shatian-SC15’ contained four and five alcohols, respectively. ‘Shatian-GX15’ and ‘Shatian-GX5’ were only found to have two and three alcohols. These indicated that cultivation region affected the alcohol biosynthesis in the ‘Shatian’ cultivar pummelo fruits. Compared to the ‘Shatian’ cultivar, the ‘Guanxi’ cultivar samples all contained pentanol, *Z*-2-penten-1-ol, hexanol and *Z*-3-hexen-1-ol. Besides, the ‘Guanxi-GD15’ and ‘Guanxi-FJ15’ samples were found to have 1-hexanol,2-ethyl, whereas octanol was only present in the ‘Guanxi-FJ15’ fruit.

Pentanol appeared to be the major alcohol in the ‘Guanxi’ cultivar, and the samples from the different regions showed a significant difference of its concentration (Table 2). For example, ‘Guanxi-GD15’ exhibited the highest pentanol level, followed by ‘Guanxi-SC15’ and then ’Guanxi-FJ15’. Meanwhile, the ‘Guanxi-FJ5’ fruit showed higher pentanol concentration than the ‘Guanxi-FJ15’ sample. These ‘Guanxi’ cultivar fruits also contained *Z*-2-penten-1-ol, hexanol and *Z*-3-hexen-1-ol as the major alcohols. The highest concentrations of these three alcohols were also found in the ‘Guanxi-GD15’ sample. Besides, the ‘Guanxi-SC15’ fruit showed a higher level of hexanol, but a lower level of *Z*-3-hexen-1-ol compared with the ‘Guanxi-FJ15’ fruit. The concentration of *Z*-2-penten-1-ol was higher in the ‘Guanxi-FJ5’ fruit than the ‘Guanxi-FJ15’ sample, whereas these Guanxi region cultivated pummelo fruits contained similar content of hexanol and *Z*-3-hexen-1-ol. In the ‘Shatian’ cultivar fruits, ‘Shatian-GD15’ and ‘Shatian-SC15’ possessed a higher content of pentanol, whereas this alcohol did not exist at a low level in the pummelo fruits cultivated in the Guangxi regions (Table 2). *Z*-2-Penten-a-ol appeared to be the highest in the ‘Shatian-SC15’ sample, followed by ‘Shatian-GD15. However, this alcohol did not exist in the Guanxi harvested pummelo samples. Hexanol was the only alcohol that was present in all of the ‘Shatian’ cultivar samples, and the cultivar cultivated in the Guangdong and Sichuan regions contained much higher content of hexanol than that from the Guangxi region. 1-Octen-3-ol and hexanol, 2-ethyl were found in the ‘Shatian-SC15’ and ‘Shatian-GX15’ samples, respectively. Their content was at a low level compared to the other alcohols found in these samples.

#### 2.2.3. Ketones

The ‘Shatian’ cultivar samples, except for ‘Shatian-SC15’, only contained one ketone (methyl isobutyl ketone). However, there were three ketones that were found in the ‘Guanxi’ cultivar samples except for 1-penten-3-one not being present in ‘Guanxi-FJ5’ (Table 2). These indicated that the pummelo cultivar genotype resulted in such a difference on the ketone composition between these two cultivars. It has been reported that pummelo fruits were not rich in ketones [2,4]. Our results were consistent with these reports [2,6]. In the ‘Shatian’ cultivar, methyl isobutyl ketone appeared to be the major ketone in these samples from the different regions. It was observed that the ‘Shatian-GD15’ and ‘Shatian-SC15’ showed higher concentration of methyl isobutyl ketone than the ‘Shatian-FJ15’ (Table 2). Meanwhile, the ‘Shatian-FJ5’ sample also contained higher concentration of methyl isobutyl ketone compared with the ‘Shatian-FJ15’. Additionally, the ‘Shatian-SC15’ sample contained 1-penten-3-one and 5-hepten-2-one and 6-methyl with a moderate and low level, respectively. In the ‘Guanxi’ cultivar, although 1-penten-3-one was not present in the ‘Guanxi-FJ5’ sample, this ketone appeared to be the main ketone in this cultivar. The highest concentration of 1-penten-3-one was observed in ‘Guanxi-GD15’, followed by the ‘Guanxi-FJ15’ and then ‘Guanxi-SC15’. Methyl isobutyl ketone was also a major ketone in the ‘Guanxi’ cultivar. However, no significant differences in its concentration were observed among these different regions’ harvested samples. 5-Hepten-2-one,6-methyl showed a higher level (about two-times higher) in ‘Guanxi-SC15’ than that in ‘Guanxi-FJ15’ and ‘Guanxi-GD15, although this ketone was not at a high level, as the other ketones in this cultivar. In addition, ‘Guanxi-FJ15’ contained a higher level of 5-hepten-2-one,6-methyl than ‘Guanxi-FJ5’.

#### 2.2.4. Esters

It has been confirmed that esters play important roles in contributing to the fruity, floral and sweet flavor notes of the overall aroma of fruits [8]. In the present study, the ester composition was displayed differently in these two pummelo cultivars. More importantly, the profile of esters in each cultivar was also altered with the different cultivation regions. For example, ‘Shatian-GD15’ and ‘Shatian-GX15’ only contained one ester (ethyl acetate), whereas four esters were found in ‘Shatian-SC15’. The ‘Shatian-GX5’ sample was found to contain six esters. In the ‘Guanxi’ cultivar, ‘Guanxi-FJ15’ exhibited four ester compounds, whereas three esters were present in ‘Guanxi-GD15’ and ‘Guanxi-FJ5’. ‘Guanxi-SC15’ only contained two esters.

Ethyl acetate appeared to be the major ester in the ‘Shatian’ cultivar. Its concentration was significantly higher (about four-times higher) in ‘Shatian-GX15’ and ‘Shatian-GD15’ compared with ‘Shatian-SC15’ (Table 2). ‘Shatian-GX15’ was about 100-times higher in the concentration of ethyl acetate than ‘Shatian-GX5’. Additionally, the concentrations of butyl acetate, ethyl octanoate and ethyl decanoate were moderate in ‘Shatian-SC15’, whereas the ‘Shatian-GX5’ sample contained a moderate level of ethyl octanoate, ethyl decanoate, butyl butanoate and 2-methyl-,2,2-dimethyl-1-(2-hydroxy-1-methylethyl) propyl propanoate. In the ‘Guanxi’ cultivar samples, the ‘Guanxi-GD15’ and ‘Guanxi-FJ15’ exhibited a higher concentration of butyl butanoate, whereas ethyl acetate was the major ester in ‘Guanxi-SC15’. Ethyl decanoate was only found in the ‘Guanxi’ sample cultivated in the Fujian region, and its concentration was moderate.

#### 2.2.5. Terpenes

Terpenes have been reported to provide fruits with a floral and fruity aroma, and more than 50 terpene compounds have been identified in fruits as the major terpenes [4,12,23]. Although terpenes have been reported to exist in fruits at a relatively low level, their low odor threshold makes these volatiles the key aromatic compound for the determination of typical fruit flavor features [8]. In the present study, ‘Shatian-SC15’ had the most complicated terpene composition (15 terpenes). However, the ‘Shatian-FJ15’ and ‘Shatian-FJ5’ samples only contained four and three terpenes, respectively (Table 2). There were eight terpenes found in ‘Shatian-GD15’. In the ‘Guanxi’ cultivar, eight terpenes were detected in the ‘Guanxi-SC15’ and ‘Guanxi-FJ15’ samples. ‘Guanxi-FJ5’ and ‘Guanxi-GD15’ only contained six and five terpenes, respectively.

Regarding the individual terpenes, limonene appeared to be the major terpene in the ‘Shatian’ cultivar samples, and its concentration displayed the regional characteristics. For example, the concentration of limonene in the ‘Shatian-GD15’ and ‘Shatian-SC15’ was about six-times higher than that in the ‘Shatian’ samples from the Guangxi region (Table 2). Linalool was also present in all of the ‘Shatian’ samples at a moderate level. However, its concentration was also much higher in ‘Shatian-SC15’ and ‘Shatian-GD15’ than ‘Shatian-GX15’ and ‘Shatian-GX5’. (*E*)-carveol also exhibited a different concentration in the different regions of the cultivated ‘Shatian’ pummelo samples. Additionally, ‘Shatian-SC15’ was totally different from the other ‘Shatian’ samples, since it contained a moderate level of β-myrcene, β-elemene, (−)-germacrene D, *cis*-linalool oxide, terpinen-4-ol, neral and citral (Table 2). In the ‘Guanxi’ cultivar, *cis*-linalool oxide, β-myrcene and limonene were the major terpenes. ‘Guanxi-GD15’ showed the highest concentration of β-myrcene and limonene, whereas the highest *cis*-linalool oxide was found in ‘Guanxi-SC15’. *trans*-Linalool oxide was also found at a high level in the ‘Guanxi’ cultivar samples, except for ‘Guanxi-GD15’. Terpinolene and terpinen-4-ol were found at a moderate concentration in ‘Guanxi-SC15’ and ‘Guanxi-FJ15’, respectively. These ‘Guanxi’ pummelo samples from the different cultivation regions contained a similar concentration of linalool (Table 2).

#### 2.2.6. Other Volatiles

‘Shatian-GD15’ only contained benzene,1-methyl-2-(1-methylethyl), whereas toluene was only present in ‘Shatian-SC15’ and ‘Shatian-GX15’ (Table 2). ‘Shatian-GX5’ was not found to have any other volatiles. In the ‘Guanxi’ cultivar, there were two other volatile compounds found in ‘Guanxi-GD15’, whereas ‘Guanxi-SC15’ only contained toluene. The ‘Guanxi’ samples from the Fujian region did not contain any other volatile compounds. Toluene appeared to be higher in ‘Shatian-SC15’ than ‘Shatian-GX15’.

### 2.3. Feature Aroma

The odor activity value (OAV) refers to the ratio calculated using the concentration of a volatile compound in fruits over its sensory threshold in water [24]. The OAV of a volatile compound in fruits can be used to indicate the sensory contribution of this volatile to the overall aroma of fruits [24]. A volatile compound in fruits with its OAV above one indicates that the flavor notes this volatile has can be effectively incorporated into the overall aroma of fruits [12]. Table 3 lists the volatile compounds that had a concentration higher than their threshold in these two pummelo cultivars. Aldehydes appeared to play more important roles in contributing their flavor notes to the overall aroma in the ‘Guanxi’ cultivar than the ‘Shatian’ cultivar. For example, hexanal has been reported to possess the grass, tallow and fat scents [3,8]. This aldehyde showed significantly higher OAV value in ‘Shatian-GD15’ and ‘Shatian-SC15’ compared to the ‘Shatian’ samples from the Guanxi region, indicating that its flavor features could be more incorporated in the ‘Shatian’ sample from the region of Guangdong and Sichuan. Meanwhile, hexanal also played a primary role in contributing its scent notes to the overall aroma of the ‘Guanxi’ pummelo cultivar due to its high OAV value. Heptanal has been described as the fat, citrus and rancid aroma [23], and the ‘Guanxi’ samples from the Fujian region could only carry its flavor notes since its OAV value was only higher than one in ‘Guanxi-FJ15’ and ‘Guanxi-FJ5’ (Table 3). 2-Hexenal has been incorporated in fruits with the apple and green aromas [23]. Its OAV value was above one in the ‘Guanxi-GD15’, ‘Guanxi-SC15’ and Guanxi-FJ15’, indicating that these ‘Guanxi’ samples possessed the obvious apple and green aromas. It has been reported that nonanal can provide fruits with the fat, citrus and green flavor notes [11]. In the present study, ‘Shatian-GD15’ and ‘Shatian-GX5’ did not have the aroma contribution from nonanal due to their concentration being below its threshold. It should be noted that the green, nut and fat notes contributed by *E*-2-octenal could be part of the overall aroma in the ‘Guanxi’ pummelo fruits since the OAV value of this volatile was higher than one in these ‘Guanxi’ samples. Similarly, the ‘Guanxi’ cultivar also exhibited the orris, fat and cucumber aroma derived from the presence of *E*-2-nonenal (Table 3). Hexanol appeared to be one of the primary alcohols that contributes its flavor notes to the pummelo overall aroma due to its high OAV value in these samples (except for ‘Shatian-GX15’) (Table 3). It has been reported that hexanol is described as the green scent [23]. Additionally, *Z*-3-hexen-1-ol provides fruits with the grass flavor [25]. This alcohol concentration in these samples was higher than its threshold except for the ‘Shatian-GX15’, indicating that its flavor characteristics could be included in the overall aroma of pummelo fruits, especially ‘Guanxi’ pummelo fruits. It should be worth noting that ‘Shatian-SC15’ was the only sample that contained 1-octen-3-ol with much higher OAV, indicating that the mushroom flavor could be present in the ‘Shatian-SC15’ pummelo fruit (Table 3). It was observed that 1-penten-3-one exhibited a higher OAV value in ‘Shatian-SC15’ than in all of the ‘Shatian’ cultivar samples, whereas the ‘Guanxi’ cultivar samples except for ‘Guanxi-FJ5’ had a concentration extremely higher than its threshold (Table 3). These indicated that 1-penten-3-one played an important role in contributing its fish and pungent flavor to the overall aroma of these samples [23]. In addition, the OAV value of ethyl acetate was higher than one in the ‘Shatian-GD15’ and ‘Shatian-GX15’ (Table 3), indicating that these pummelo fruits possessed the pineapple flavor that was derived from ethyl acetate [26]. Ethyl octanoate has been described as the fruity and fat flavor notes [6], and its scent features were incorporated in ‘Shatian-SC15’ and ‘Shatian-GX5’ due to their high OAV value. The typical aroma that butyl butanoate provides could also be present in the ‘Guanxi’ cultivar samples since these samples harvested from the different regions had a concentration above its odor threshold (Table 3). Regarding the terpenes, limonene and linalool were considered the major volatile compounds that could provide the featured aromas to the ‘Shatian’ cultivar, whereas the overall aroma of the ‘Guanxi’ cultivar was mainly comprised of β-myrcene, limonene, *cis*-linalool oxide and linalool (Table 3). Limonene has been reported to possess the citrus and mint flavor, whereas linalool has been described as the flower and lavender scents [5]. β-Myrcene has been reported to provide fruits with the balsamic, must and spice flavors, and the featured aroma *cis*-linalool oxide brings is the flower fragrance [5]. Besides, the turpentine, nutmeg and must aromas might be present in ‘Shatian-SC15’ and ‘Guanxi-FJ15’ since the OAV value of terpinen-4-ol in these samples was above one (Table 3). Citral has been confirmed to show the lemon aroma [5], and its aroma could be significantly improved in the ‘Shatian’ cultivar from the Guangdong and Sichuan regions. Similarly, the flower note derived from geranylacetone could be incorporated effectively in ‘Shatian-GD15’, ‘Guanxi-GD15 and ‘Guanxi-FJ15’.

### 2.4. Multivariate Analysis

#### 2.4.1. Cluster Analysis

**Figure 1:**
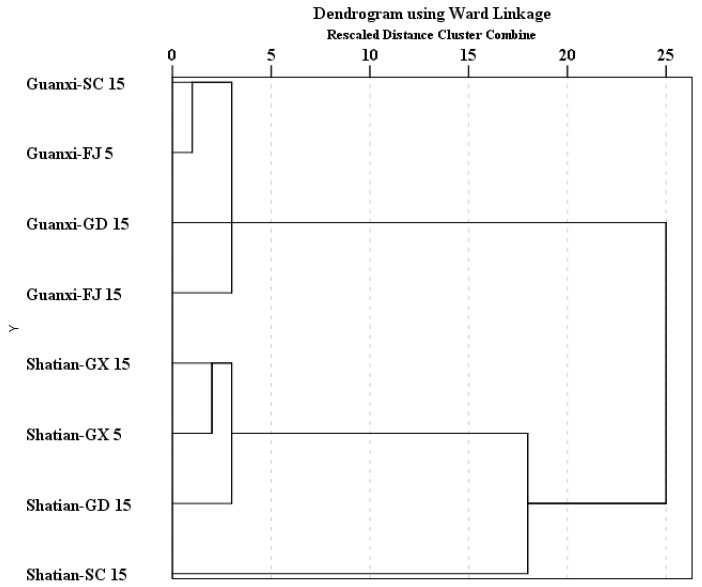
Cluster analysis of the volatile profile in two pummelo cultivars cultivated in different regions of China.

Cluster analysis was carried out using all of the detected volatile compounds as the variable to better understand the volatile profile similarity in these two pummelo cultivar samples cultivated from different regions of China (Figure 1). It was obviously observed that all of the ‘Guanxi’ cultivar samples were grouped together, whereas all of the ‘Shatian’ samples were assembled. A significant difference in the volatile profile was observed between the ‘Guanxi’ and ‘Shatian’ cultivars. These indicated that cultivar genotype appeared to play a primary role in the determination of the volatile profile in pummelo cultivars. However, it should be made known that the ‘Shatian-SC15’ sample in the ‘Shatian’ sample group exhibited a long distance compared with the other ‘Shatian’ samples on the cluster scale. This indicated that the regional characteristics also could regulate the accumulation of volatile compounds in fruits, which could further affect the volatile profile in pummelo cultivars. Additionally, an obvious difference on the volatile profile was observed between ‘Shatian-GX15’ and ‘Shatian-GX5’. However, ‘Guanxi-FJ15’ exhibited a similar volatile profile to ‘Guanxi-FJ5’. These indicated that tree age might also exert an effect on the alteration of the volatile composition in pummelo fruits. However, such an effect might be a cultivar-dependent effect.

#### 2.4.2. Principal Component Analysis

**Figure 2:**
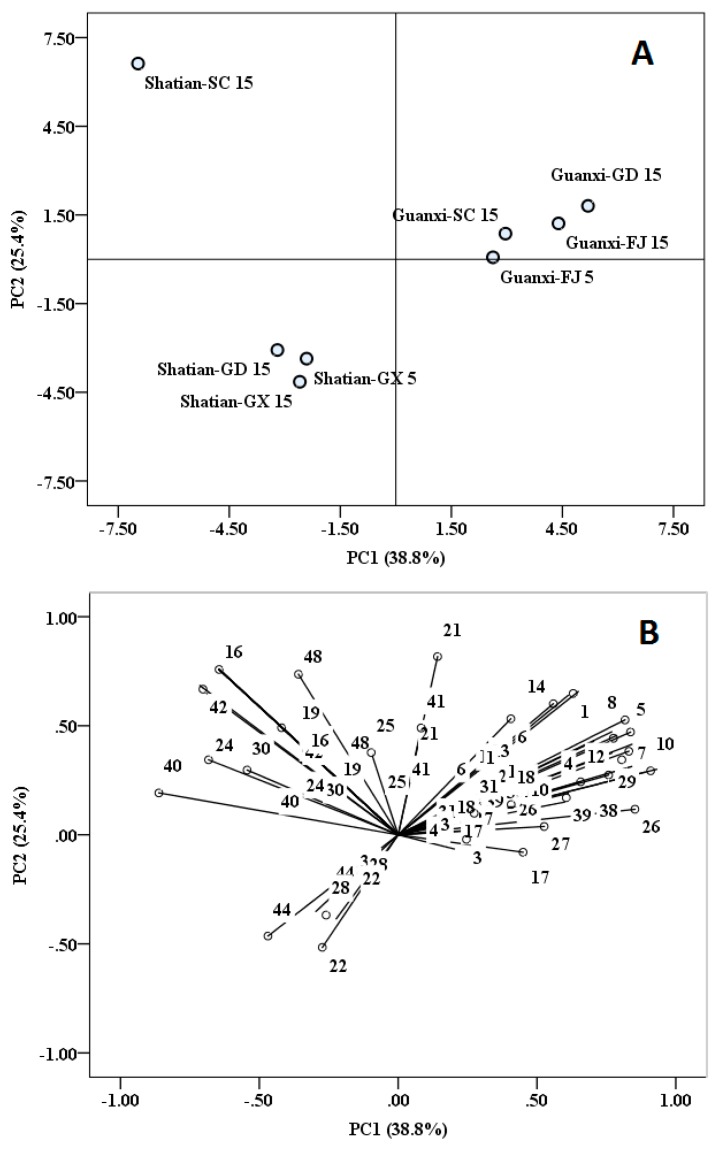
(**A**) Score plot and (**B**) loading plot of principal component analysis of the volatile profile in two pummelo cultivars cultivated from different regions of China. The number of volatile compounds corresponds with Supplementary Table S2.

Principal component analysis was also performed in the present study using the detected volatile compounds as the variables. This multivariate analysis also could elucidate the similarity of the volatile profiles in these cultivars from different cultivation regions and further screen out the key volatile compounds that play important roles in separating these cultivar samples (Figure 2).

The first and second principal components (PC1 and PC2) explained about 38.6% and 25.0% of the total variances (Figure 2A). In the score plot, it was clearly observed that all of the ‘Guanxi’ cultivar samples cultivated from different regions were aggregated together, whereas ‘Shatian-GX15’, ‘Shatian-GX5’ and ‘Shatian-GD15’ were assembled together. Additionally, these two cultivar samples were segregated from each other. These results indicated that the cultivar genotype was the main factor that resulted in the volatile profile difference in different pummelo cultivars. However, the ‘Shatian-SC15’ sample was positioned at the left side of the PC1 scale and the top scale of the PC2, which resulted in this sample being far away from the other ‘Shatian’ cultivar samples. This was similar to the result from the cluster analysis (Figure 1).

In Figure 2B (loading plot), it was observed that linalool (No. 40), α-terpineol (No. 42) and ethyl octanoate (No. 24) were negatively correlated with PC1. Meanwhile, the volatile compounds that significantly exerted a positive correlation with PC1 included butyl butanoate (No. 26), benzaldehyde (No. 10), *E*,*E*-2,4-heptadienal (No. 9), *Z*-2-heptenal (No. 5), *E*-2-pentenal (No. 2) and pentanol (No. 12). These volatile compounds were screened out to be the key volatiles that separate the ‘Shatian’ and ‘Guanxi’ pummelo cultivars. For example, *E*-2-pentenal, *Z*-2-heptenal, *E*,*E*-2,4-heptadienal and benzaldehyde did not exist or existed in a low amount in the ‘Shatian’ cultivar samples from the different regions. However, the ‘Guanxi’ cultivar samples were rich in these aldehydes. The significant concentration differences on pentanol between these two cultivars also played a primary role in differentiating the ‘Shatian’ and ‘Guanxi’ cultivars. Besides, (*E*)-carveol (No. 44), β-neoclovene (No. 37), 2-methyl-,2,2-dimethyl-1-(2-hydroxy-1-methylethyl) propyl propanoate (No. 28) and ethyl acetate (No. 22) were positioned at the negative scale of PC2, indicating that these volatiles were negatively correlated with PC2. However, the volatile compounds positively correlated with PC2 were found to be 1-octen-3-ol (No. 16), toluene (No. 48) and α-terpineol (No. 42). The differences of these volatile compounds’ concentration resulted in the segregation of ‘Shatian-SC15’ from the other ‘Shatian’ samples, indicating that regional environment could affect the volatile biosynthesis in the same pummelo cultivar.

### 2.5. Regional Characteristics

It has been accepted that cultivation region affects the accumulation of secondary metabolites in fruits since different climates and environmental features could regulate the expression and activation of genes and enzymes that determine the biosynthesis of secondary metabolites [12,15,16]. Regarding cultivation regions of pummelo fruits in China, the Guangdong region is in the south of China. Similarly, another region for the pummelo cultivation in the south of China is Guangxi. These two regions exhibit the subtropical monsoon climate, and their average annual precipitation is about 1500 mm. Besides, sufficient temperature and sunshine duration in these two regions have positive effects on the accumulation of secondary metabolites, such as volatile compounds [15]. The Sichuan cultivation region is in the Sichuan basin, in the southwest part of China, with a subtropical humid monsoon climate feature. Compared to the Guangdong and Guangxi regions, the annual rainfall amount in the Sichuan cultivation region is much less. The Fujian region belongs to the southeast part of the pummelo cultivation area in China, and this region shares similar climate features as the Guangdong and Guangxi regions. The soil types in the Guangdong, Guangxi, Fujian and Sichuan region are red earths, acid purplish soils, lateritic red earths and calcic purplish soils, respectively. From the cluster and principal component analysis, a similar volatile profile was observed in the ‘Guanxi’ cultivar samples cultivated in the Guangdong, Sichuan and Fujian regions. No significant differentiation of the volatile profile was observed in ‘Guanxi-FJ15’ and ‘Guanxi-FJ5’. However, a significant difference of the volatiles concentration in these two pummelo samples was observed (Table 2), indicating that tree age might also affect the volatile accumulation. Similarly, ‘Shatian-GX15’ showed a similarity in the volatile profile with ‘Shatian-GX5’. However, the difference of the individual volatiles’ concentration in these two pummelo samples indicated that the biosynthesis of volatile compounds in the ‘Shatian’ cultivar might also be impacted by tree age. However, an obvious separation of ‘Shatian-SC15’ from the other ‘Shatian’ cultivar samples was observed, which might mainly result from the obvious climate difference in the Sichuan region compared to the Guangdong and Guangxi regions. This result was also consistent with our previous report where the phenolic profile in the ‘Shatian’ cultivar in the Sichuan region was different from that in the Guangdong and Guangxi regions [16].

## 3. Experimental Section

### 3.1. Chemicals and Standards

Volatile compound standards, including hexanal, 2-hexenal, nonanal, benzaldehyde, pentanol, hexanol, *Z*-3-hexen-1-ol, 1-octen-3-ol, 1-hexanol,2-ethyl-, octanol, *p*-cymene, 2-heptanone, ethyl acetate, butyl acetate, ethyl octanoate, ethyl decanoate, β-myrcene, limonene, terpinolene, α-lonone, *cis*-linalool oxide, linalool, α-terpineol, geraniol, citral and geranylacetone, were purchased from Sigma-Aldrich (St. Louis, MO, USA). These standards had a purity above 95%. The detailed information of these volatile standards is listed in Supplementary Table S1. Ethanol (99.99%) was purchased from Sigma-Aldrich (St. Louis, MO, USA). Citric acid and glucose were received from Sinopharm Chemical Reagent Beijing Co., Ltd (Beijing, China). NaCl was purchased from the Tianjin Chemical Reagent Plant (Tianjin, China). Milli-Q water was purified from a Milli-Q purification system (Millipore, Bedford, MA, USA).

### 3.2. Pummelo Fruits

Two pummelo cultivars were selected in the present study, including the ‘Shantian’ and ‘Guanxi’ pummelo cultivars. In the ‘Shatian’ pummelo cultivar, the pummelo fruits were harvested from Dapu of Guangdong, Nanbu of Sichuan and Pingle of Guangxi on 15-year old pummelo trees (Shatian-GD15, Shatian-SC15 and Shatian-GX15, respectively). The other ‘Shatian’ pummelo cultivar sample was also harvested in 2009 from Pingle of Guangxi region on 5-year old pummelo trees (Shatian-GX5). Regarding the ‘Guanxi’ cultivar, the fruits were harvested from the region of Dapu in Guangdong, Nanbu in Sichuan and Pinghe in Fujiang on 15-year old pummelo trees (Guanxi-GD15, Guanxi-SC15 and Guanxi-FJ15, respectively). The Guanxi-FJ5 pummelo fruits were harvested also from the region of Pinghe in Fujiang on 5-year old trees. The detailed conditions of these cultivation regions were introduced in our previous study [16]. Regarding sample harvesting for each cultivar, three individual trees were randomly selected in the same orchard. Afterwards, three fully-ripened pummelo fruits, with one at the top of the tree, one in the mid-section of the tree and the other one at the bottom of tree, were randomly picked from each tree. After harvest, the pummelo fruit samples were immediately transported to our lab. The pummelo fruits were weighed firstly and then peeled to obtain flesh and flavedo. The flavedo parts were discarded, whereas the flesh of the three pummelo fruits (n = 3) was combined and immediately frozen using liquid nitrogen. Afterwards, the frozen flesh was crushed and then thawed into the raw juice. The raw juice of each sample was centrifuged at 4000 rpm for 10 min to collect the juice for the measurement of their total soluble solids, total sugar and total acidity (Table 1).

### 3.3. Head Space Solid Phase Microextraction

Head space solid phase microextraction of volatile compounds from the pummelo juice followed a published method with minor modifications [27]. In brief, the pummelo juice (5.0 mL), 10 µL of 2.004 mg/L methyl-2-pentanol solution (internal standard) and 1.0 g of sodium chloride were mixed into a 15-mL vial that contained a magnetic stirrer. Subsequently, the vial was immediately capped with a PTFE-silicon septum, followed by being equilibrated for 30 min at 40 °C under agitation. After the equilibration, an activated 50/30-µm Divinylbenzene/Carboxen/Polydim--ethylsiloxane carboxen fiber (Supelco, Bellefonte, PA, USA) was immediately inserted into the head space of the vial, and the head space microextraction was carried out for 30 min at 40 °C under continuous agitation. Finally, the fiber was removed from the head space of the vial and immediately inserted into the injector of the GC for desorption of volatile compounds at 250 °C for 5 min. The head space solid phase microextraction of each juice sample was carried out in triplicate.

### 3.4. GC-MS Analysis

GC-MS analysis of volatile compounds in the pummelo juice also followed a published method with minor modifications [27]. The volatile compounds were analyzed using an Agilent 6890 Gas Chromatography (GC) coupled with an Agilent 5975 Mass Spectrometer (MS) (Agilent Technologies, Santa Clara, CA, USA). An HP-INNOWAX 60 m × 0.25 mm × 0.25 µm column (J&W Scientific, Folsom, CA, USA) was used to separate these volatile compounds. The carrier gas (helium) was set at a 1-mL/min flow rate under a splitless inlet mode. The oven temperature gradient was programmed as follows: 40 °C for 2 min, then increased to 200 °C at a rate of 3 °C /min and kept at 200 °C for 2 min. The mass spectrometer in the electron impact mode (MS/EI) at 70 eV scanned in the range of *m*/*z* 20–450. The mass spectrometer was operated in full scan for the qualitative analysis and selective ion mode (SIM) for the quantitative analysis under auto-tune conditions at the same time. For the volatiles with the reference standard, the identification of volatile compounds was carried out by comparing their mass spectrum and retention index with the retention index and mass spectrum of the corresponding standard and further confirmed using the NIST08 standard reference library. Regarding the volatile quantitation, a synthetic juice matrix, consisting of 100 g/L glucose, 5 g/L citric acid, 1% ethanol and pH 3.5, was prepared, and all of the volatile standards with seven successive concentrations were dissolved in this matrix with the internal standard. The same head space microextraction was performed in the matrix. The volatiles with the available standard were quantified using the peak area ratio of the standard over the internal standard versus the standard concentration. For the volatiles without the reference standard, they were quantified using a standard similar to their chemical structure or carbon atom numbers. The detailed information of the volatile quantitation is listed in Supplementary Table S2.

### 3.5. Statistical Analyses

Data were expressed as the mean ± standard deviation of triplicate tests. One-way analysis of variance (ANOVA) was used to compare the significant differences among the means using SPSS Version 19.0 under the Duncan test at a *p*-level of 0.05 (SPSS Inc., Chicago, IL, USA). Principal component analysis was used to elucidate the similarity of the volatile profile in these pummelo juices using the detected volatile compounds as the variables (scaled) under SPSS Version 19.0. Hierarchical cluster analysis was also carried out using the detected volatiles as variables. Ward’s method was used as the linkage rule, whereas the squared Euclidean distance was taken as a measure of the proximity between two pummelo samples.

## 4. Conclusions

In conclusion, the ‘Guanxi’ cultivar possessed a more complex composition of volatile compounds compared to the ‘Shatian’ cultivar. Meanwhile, the ‘Guanxi’ cultivar contained a higher level of volatile compounds than the ‘Shatian’ cultivar. Multivariate analyses, including cluster and principal component analysis, revealed that pummelo cultivar genotype played a primary role in differentiating the volatile profile in the pummelo cultivar, whereas cultivation features also affected the accumulation of volatile compounds in pummelo.

## Figures and Tables

**Table 1:** Fruit weight, total soluble solids, total sugar and total acidity in two pummelo cultivars cultivated in different regions of China.

Pummelo Cultivar	Fruit Weight (kg)	Total Soluble Solids (%)	Total Sugar (g Glucose/L)	Total Acidity (g Citric Acid/L)
Shatian-GD15	1.1 ± 0.0 a	16.1 ± 0.4 a	111.7 ± 8.1 a	2.6 ± 0.1 d
Shatian-SC15	1.2 ± 0.1 a	10.2 ± 0.3 bc	91.3 ± 5.2 b	4.5 ± 0.2 c
Shatian-GX15	1.5 ± 0.0 a	13.1 ± 0.5 b	111.2 ± 6.3 a	3.7 ± 0.0 c
Shatian-GX5	1.3 ± 0.1 a	12.2 ± 0.6 b	103.2 ± 4.5 ab	2.4 ± 0.1 d
Guanxi-GD15	2.4 ± 0.0 b	12.0 ± 0.3 b	109.0 ± 2.3 ab	6.6 ± 0.1 b
Guanxi-SC15	2.3 ± 0.1 b	12.4 ± 0.4 b	112.5 ± 3.9 a	10.6 ± 0.6 a
Guanxi-FJ15	2.5 ± 0.1 b	12.1 ± 0.6 b	104.6 ± 4.1 ab	11.6 ± 1.0 a
Guanxi-FJ5	2.4 ± 0.1 b	12.8 ± 0.3 b	98.6 ± 7.3 b	9.1 ± 0.2 a

Data are the mean ± standard deviation of triplicate tests. Different letters in each column indicate significant differences at a significance level of 0.05.

**Table 2:** Individual volatile content in two pummelo cultivars harvested from different regions of China.

Volatile Compound (µg/L)	No.	Shatian-GD15	Shatian-SC15	Shatian-GX15	Shatian-GX5	Guanxi-GD15	Guanxi-SC15	Guanxi-FJ15	Guanxi-FJ5
***Aldehydes***									
Hexanal	1	5369.6 ± 62.4 d	7401.4 ± 545.9 cd	679.7 ± 18.4 e	801.1 ± 34.4 e	13388.3 ± 3094.1 a	8541.5 ± 440.5 c	7512.5 ± 267.0 c	9700.6 ± 229.7 bc
*E*-2-Pentenal	2	nd	244.9 ± 21.9 d	nd	nd	1527.2 ± 24.5 a	817.9 ± 137.5 c	837.3 ± 78.2 c	1215.9 ± 71.5 b
Heptanal	3	nd	nd	nd	nd	nd	nd	27.8 ± 0.0 b	256.3 ± 3.5 a
*E*-2-Hexenal	4	nd	nd	nd	nd	58.7 ± 0.0 b	25.3 ± 0.2 c	136.9 ± 1.1 a	nd
*Z*-2-Heptenal	5	16.1 ± 0.0 d	284.2 ± 21.6 c	nd	nd	1411.5 ± 0.5 a	778.7 ± 101.1 b	782.7 ± 7.0 b	862.3 ± 0.9 b
Nonanal	6	nd	129.7 ± 10.1 a	139.7 ± 5.3 a	nd	136.7 ± 4.2 a	131.3 ± 0.5 a	136.0 ± 3.0 a	128.7 ± 1.1 a
*E*,*E*-2,4-Hexadienal	7	nd	61.8 ± 5.5 d	nd	nd	594.2 ± 84.3 a	235.8 ± 28.2 c	264.8 ± 10.7 bc	355.9 ± 13.8 b
*E*-2-Octenal	8	nd	124.2 ± 69.1 c	nd	15.9 ± 1.6 d	332.7 ± 67.2 ab	372.3 ± 50.6 a	312.8 ± 2.2 ab	268.9 ± 26.3 b
*E*,*E*-2,4-Heptadienal	9	nd	2.9 ± 0.1 d	nd	nd	55.9 ± 1.2 b	90.1 ± 20.5 a	58.1 ± 4.2 b	42.0 ± 6.4 bc
Benzaldehyde	10	nd	nd	nd	nd	76.1 ± 0.0 a	74.6 ± 0.9 a	75.9 ± 0.7 a	75.5 ± 0.0 a
*E*-2-Nonenal	11	nd	nd	nd	nd	17.1 ± 4.9 a	26.2 ± 12.9 a	25.5 ± 0.3 a	18.7 ± 0.7 a
***Alcohols***									
Pentanol	12	1115.6 ± 84.3 d	1240 ± 12.1 d	nd	404.0 ± 48.9 e	3384.4 ± 333.7 a	2547.9 ± 119.2 b	2058.3 ± 153.1 c	3097.7 ± 71.4 a
*Z*-2-Penten-1-ol	13	399.7 ± 2.0 d	1016.9 ± 175.3 c	nd	nd	1915.6 ± 80.5 a	998.0 ± 98.3 c	1202.1 ± 124.6 c	1560.6± 9.3 b
Hexanol	14	912.0 ± 2.0 c	979.1 ± 46.5 c	242.2 ± 0.0 e	527.5 ± 95.1 d	1523.8 ± 174.4 a	1316.6 ± 73.0 b	939.0 ± 11.7 c	991.2 ± 6.3 c
*Z*-3-Hexen-1-ol	15	279.7 ± 91.4 e	516.9 ± 56.6 d	nd	345.1 ± 78.9 de	1959.9 ± 176.8 a	809.4 ± 97.1 c	1087.4 ± 20.4 b	1154.9 ± 32.9 b
1-Octen-3-ol	16	nd	39.7 ± 0.4	nd	nd	nd	nd	nd	nd
1-Hexanol, 2-ethyl-	17	nd	nd	31.3 ± 0.2 a	nd	34.1 ± 0.2 a	nd	29.7 ± 0.9 a	nd
Octanol	18	nd	nd	nd	nd	nd	nd	13.6 ± 0.2	nd
***Ketones***									
Methyl isobutyl ketone	19	1502.3 ± 71.9 ab	1615.6 ± 752.3 ab	523.3 ± 12.4 c	1216.9 ± 292.7 b	1053.9 ± 39.9 b	1057.3 ± 7.4 b	1075.6 ± 17.8 b	1057.4 ± 38.5 b
1-Penten-3-one	20	nd	661.0 ± 0.3 d	nd	nd	4252.3 ± 290.3 a	1850.9 ± 10.1 c	2507.3 ± 99.4 b	nd
5-Hepten-2-one, 6-methyl-	21	nd	57.8 ± 1.1 a	nd	nd	23.7 ± 2.8 b	64.9 ± 6.4 a	36.5 ± 0.2 b	15.4 ± 0.9 c
***Esters***									
Ethyl acetate	22	19,117.8 ± 758.0 b	500.4 ± 226.4 e	203,729.4 ± 73.6 a	2326.2 ± 309.8 c	102.4 ± 1.4 g	969.2 ± 37.7 d	288.5 ± 11.1 f	429.7 ± 73.7 e
Butyl acetate	23	nd	413.4 ± 13.6	nd	nd	nd	nd	nd	nd
Ethyl octanoate	24		297.2 ± 2.3 a		259.1 ± 2.1 a				
Ethyl decanoate	25	nd	592.4 ± 6.9 b	nd	544.1 ± 2.3 b	nd	nd	714.3 ± 0.7 a	514.5 ± 16.2 c
Butyl butanoate	26	nd	nd	nd	620.4 ± 32.1 b	939.7 ± 90.3 a	488.6 ± 4.4 b	871.5 ± 31.8 a	582.4 ± 146.9 b
Isobutyl 2,2,4-trimethyl-3-carboxyisopropyl pentanoate	27	nd	nd	nd	478.1 ± 4.4 b	642.0 ± 6.5 a	nd	610.7 ± 6.1 a	nd
2-Methyl-, 2,2-dimethyl-1-(2-hydroxy-1-methylethyl), propyl propanoate	28	nd	nd	nd	485.1 ± 19.2	nd	nd	nd	nd
***Terpenes***									
β-Myrcene	29	nd	330.9 ± 11.4 d	nd	nd	2760.3 ± 898.9 a	451.7 ± 76.9 cd	1394.1 ± 282.4 bc	1631.9 ± 754.7 bc
Limonene	30	3480.6 ± 498.9 a	2935.1 ± 29.6 b	572.4 ± 78.3 d	598.6 ± 6.6 d	1545.5 ± 190.5 c	1008.5 ± 150.3 c	351.8 ± 85.2 d	510.0 ± 54.8 d
Terpinolene	31	nd	nd	nd	nd	nd	164.9 ± 10.3	nd	nd
β-Elemene	32	nd	249.6 ± 2.7	nd	nd	nd	nd	nd	nd
(−)-Germacrene D	33	nd	434 ± 81.2	nd	nd	nd	nd	nd	nd
α-Muurolene	34	nd	7.5 ± 0.3	nd	nd	nd	nd	nd	nd
Copaene	35	nd	10.9 ± 0.6	nd	nd	nd	nd	nd	nd
δ-Cadinene	36	nd	12.1 ± 0.0	nd	nd	nd	nd	nd	nd
β-Neoclovene	37	4.8 ± 0.3	nd	nd	nd	nd	nd	nd	nd
*cis*-Linalool oxide	38	66.3 ± 2.3 f	117.0 ± 1.2 e	nd	nd	1147.0 ± 146.1 c	3350.2 ± 596.7 a	2953.1 ± 17.1 b	1452.9 ± 264.5 c
*trans*-Linalool oxide	39	nd	nd	nd	nd	nd	986.1 ± 19.7 a	1129.6 ± 79.5 a	421.8 ± 98.3 b
Linalool	40	99.4 ± 1.3 b	117.9 ± 24.0 a	58.9 ± 1.7 c	16.8 ± 0.9 d	10.7 ± 0.9 d	7.1 ± 2.2 d	7.6 ± 1.7 d	18.5 ± 0.1 d
Terpinen-4-ol	41	nd	169.3 ± 1.7 b	nd	nd	nd	nd	341.2 ± 3.5 a	nd
α-Terpineol	42	15.0 ± 1.4 b	60.5 ± 1.8 a	6.0 ± 0.6 c	12.6 ± 2.7 b	nd	5.2 ± 0.0 c	18.5 ± 0.0 b	nd
Geraniol	43	nd	8.4 ± 0.7	nd	nd	nd	nd	nd	nd
(*E*)-Carveol	44	244.6 ± 32.6 a	28.5 ± 4.2 c	107.3 ± 2.9 b	nd	nd	nd	nd	nd
Neral	45	nd	356.9 ± 30.7	nd	nd	nd	nd	nd	nd
Citral	46	146.6 ± 39.9 b	586.4 ± 47.3 a	nd	nd	nd	30.4 ± 0.0 c	nd	nd
Geranylacetone	47	83.3 ± 0.0 b	nd	nd	nd	158.4 ± 16.4 b		318.7 ± 30.5 a	44.5 ± 0.0 c
***Other volatiles***									
Toluene	48	nd	499.3 ± 4.1 a	158.3 ± 3.6 d	nd	226.2 ± 2.2 c	249.6 ± 12.2 b	nd	nd
Benzene, 1-methyl-2-(1-methylethyl)-	49	111.65 ± 0.0 b	nd	nd	nd	138.2 ± 45.4 b	nd	nd	nd

Data are the mean ± standard deviation of triplicate tests. Different letters in each column indicate significant differences at a significant level of 0.05. ‘nd’ represents ‘not detected’.

**Table 3:** Aroma descriptor, odor threshold and odor activity value of major volatile compounds in two pummelo cultivars cultivated in different regions of China.

Volatile Compound	No.	Aroma Descriptor	Odor Threshold (µg/L)	Odor Activity Value							
				Shatian-GD15	Shatian-SC15	Shatian-GX15	Shatian-GX5	Guanxi-GD15	Guanxi-SC15	Guanxi-FJ15	Guanxi-FJ5
***Aldehydes***											
Hexanal	1	grass, tallow, fat	4.5 [23]	1193.2 ± 13.8	1644.8 ± 121.3	151.0 ± 4.1	202.6 ± 7.6	2975.2 ± 687.6	1898.1 ± 97.9	1669.5 ± 59.3	2155.7 ± 51.0
Heptanal	3	fat, citrus, rancid	3 [25]	nd	nd	nd	nd	nd	nd	9.3 ± 0.0	85.4 ± 1.2
2-Hexenal	4	apple, green	17 [25]	nd	nd	nd	nd	3.5 ± 0.0	1.5 ± 0.0	8.1 ± 0.1	nd
Nonanal	6	fat, citrus, green	1 [25]	nd	129.7 ± 10.1	139.7 ± 5.3	0	136.7 ± 4.2	131.3 ± 0.5	136.0 ± 3.0	128.7 ± 1.1
*E*-2-Octenal	8	green, nut, fat	3 [23]	nd	41.4 ± 23.0	nd	5.3 ± 0.5	110.9 ± 22.4	124.1 ± 16.9	104.3 ± 0.7	89.6 ± 8.8
Benzaldehyde	10	almond, burnt sugar	350 [23]	nd	nd	nd	nd	0.2 ± 0.0	0.2 ± 0.0	0.2 ± 0.0	0.2 ± 0.0
*E*-2-Nonenal	11	orris, fat, cucumber	0.08 [8,23]	nd	nd	nd	nd	214.1 ± 61.3	327.9 ± 161.3	318.9 ± 3.8	233.7 ± 8.8
***Alcohols***											
Pentanol	12	fruit	4000 [23]	0.3 ± 0.0	0.3 ± 0.0	nd	0.1 ± 0.0	0.8 ± 0.1	0.6 ± 0.0	0.5 ± 0.0	0.8 ± 0.0
Hexanol	14	green	500 [23]	1.8 ± 0.0	2.0 ± 0.1	0.5 ± 0.0	1.1 ± 0.2	3.0 ± 0.3	2.6 ± 0.1	1.9 ± 0.0	2.0 ± 0.0
*Z*-3-Hexen-1-ol	15	grass	70 [25]	4.0 ± 1.3	7.4 ± 0.8	nd	5.2 ± 1.1	28.0 ± 2.5	11.6 ± 1.4	15.5 ± 0.3	16.5 ± 0.5
1-Octen-3-ol	16	mushroom	1 [25]	nd	39.7 ± 0.4	nd	nd	nd	nd	nd	nd
Octanol	18	moss, nut, mushroom	190 [23]	nd	nd	nd	nd	nd	nd	0.1 ± 0.0	nd
***Ketones***											
1-Penten-3-one	20	fish, pungent	1 [25]	nd	661.0 ± 0.3	nd	nd	4252.3 ± 290.3	1850.9 ± 10.1	2507.4 ± 99.4	nd
5-Hepten-2-one, 6-methyl-	21		50 [25]	nd	1.2 ± 0.0	nd	nd	0.5 ± 0.1	1.3 ± 0.1	0.7 ± 0.0	0.3 ± 0.0
***Esters***											
Ethyl acetate	22	pineapple	5000 [23]	3.8 ± 0.2	0.1 ± 0.0	40.7 ± 0.0	0.5 ± 0.1	<0.1	0.2 ± 0.0	0.1 ± 0.0	0.1 ± 0.0
Ethyl octanoate	24	fruit, fat	194 [23]	nd	1.4 ± 0.0	nd	1.3 ± 0.0	nd	nd	nd	nd
Ethyl decanoate	25	grape	6300 [23]	nd	0.1 ± 0.0	nd	0.1 ± 0.0	nd	nd	0.1 ± 0.0	0.1 ± 0.0
Butyl butanoate	26	fruit	400 [23]	nd	nd	nd	1.6 ± 0.1	2.3 ± 0.2	1.2 ± 0.0	2.2 ± 0.1	1.5 ± 0.4
***Terpenes***											
β-Myrcene	29	balsamic, must, spice	15 [23]	nd	22.1± 0.8	nd	nd	184.0 ± 59.9	30.1 ± 5.1	92.9 ± 18.8	108.8 ± 50.3
Limonene	30	citrus, mint	34 [8]	102.9 ± 14.7	85.3 ± 0.9	17.6 ± 2.3	17.6 ± 0.2	44.1 ± 5.6	29.4 ± 4.4	11.7 ± 2.5	14.7 ± 1.6
Terpinolene	31		200 [23]	nd	nd	nd	nd	nd	0.8 ± 0.1	nd	nd
*cis*-Linalool oxide	38	flower	320 [25]	0.2 ± 0.0	0.4 ± 0.0	nd	nd	3.6 ± 0.5	10.5 ± 1.9	9.2 ± 0.1	4.5 ± 0.8
Linalool	40	flower, lavender	6 [23]	16.6 ± 0.2	19.7 ± 4.0	9.8 ± 0.3	2.8 ± 0.2	1.8 ± 0.2	1.2 ± 0.4	1.3 ± 0.3	3.1 ± 0.0
Terpinen-4-ol	41	turpentine, nutmeg, must	130 [25]	nd	1.3 ± 0.0	nd	nd	nd	nd	2.6 ± 0.0	nd
α-Terpineol	42	oil, anise, mint	330 [25]	<0.1	0.2±	<0.1	<0.1	nd	<0.1	0.1± 0.0	nd
Geraniol	43	rose, geranium	40 [25]	nd	0.2 ± 0.0	nd	nd	nd	nd	nd	nd
Neral	45	lemon	1000 [25]	nd	0.4 ± 0.0	nd	nd	nd	nd	nd	nd
Citral	46	lemon	85 [25]	1.7 ± 0.5	6.9 ± 0.6	nd	nd	nd	0.4 ± 0.0	nd	nd
Geranylacetone	47	flower	60 [23]	1.4 ± 0.0	nd	nd	nd	2.6 ± 0.4	nd	5.3 ± 0.5	0.7 ± 0.0

Data are the mean ± standard deviation of triplicate tests. ‘nd’ represents ‘not detected’.

## Supplementary Materials

The following are available online: Table S1: Standard information; Table S2: Identification of volatile compounds in two pummelo cultivars cultivated in different regions of China.
