# Supplementary material for: Volatile Composition in Two Pummelo Cultivars (Citrus grandis L. Osbeck) from Different Cultivation Regions in China
Source: Molecules. 2017 Apr 29;22(5):716. doi: 10.3390/molecules22050716 (PMC6154625; doi:10.3390/molecules22050716)
Supplement: Supplementary file 1 [file molecules-22-00716-s001.pdf]

Supplementary Materials: Volatile Composition in Two Pummelo Cultivars (*Citrus grandis* L. Osbeck) from Different Cultivation Regions in China

Mingxia Zhang [1,*], Linbo Li [1], Zhongwei Wu [1], Yanjie Wang [1], Yiming Zang [2] and Guojie Liu [2]

Supplementary Table S1. Standard information.

Volatile Compound	Purity	Manufacturers	Calibration Curves	R^2	Linear Range (μg/L)
Hexanal	0.99	Sigma–Aldrich	y = 8,587,213x + 934,365	0.998	6.9–16,777.4
2-Hexenal	0.99	Sigma–Aldrich	y = 20,250,155x + 1,370,372	0.955	2.2–9681.6
Nonanal	0.95	Sigma–Aldrich	y = 76,857,211x−8,292,053	0.986	32.5–450.8
Benzaldehyde	0.99	Sigma–Aldrich	y = 52,203,029x + 4,808,494	0.991	2.1–2863.9
Pentanol	0.99	Sigma–Aldrich	y = 2,872,072x + 287,093	0.999	27.5–1955.2
Hexanol	0.98	Sigma–Aldrich	y = 24,539,770x + 35,235,662	0.901	2.2–9504.0
Z-3-Hexen-1-ol	0.97	Sigma–Aldrich	y = 3,006,405x + 1,043,315	0.995	10.1–1756.2
1-Octen-3-ol	0.98	Sigma–Aldrich	y = 131,651,142x + 8,150,433	0.976	0.9–517.4
1-Hexanol, 2-ethyl-	0.99	Sigma–Aldrich	y = 218,281,528x + 8,432,056	0.986	0.2–843.8
Octanol	0.99	Sigma–Aldrich	y = 37,619,883x + 1,311,491	0.928	4.4–171.9
p-cymene	0.99	Sigma–Aldrich	y = 20,000,000x−733,843	0.995	61.5–855.6
2-Heptanone	0.97	Sigma–Aldrich	y = 48,188,131x + 596,641	0.994	6.7–1906.8
Ethyl acetate	0.99	Sigma–Aldrich	y = 5,611,795x + 8,831,449	0.977	4.5–19,660.3
Butyl acetate	0.99	Sigma–Aldrich	y = 5,676,819x + 386,203	0.977	0.3–1371.8
Ethyl octanoate	0.99	Sigma–Aldrich	y = 83,578,849x−19,961,874	0.993	4.3–190,443.0
Ethyl decanoate	0.99	Sigma–Aldrich	y = 27,556,033x−12,192,555	0.998	7.1–30,733.6
β-Myrcene	0.95	Sigma–Aldrich	y = 9,181,729x + 533,729	0.998	20.8–3530.1
Limonene	0.95	Sigma–Aldrich	y = 8,379,080x + 1,567,900	0.957	20.9–3531.2.

Terpinolene	0.99	Sigma–Aldrich	y = 8,573,938x + 105,833	0.997	1.5–475.4
α-Ionone	0.99	Sigma–Aldrich	y = 254,789,828x−247,795	0.994	1.9–145.9
cis-Linalool oxide	0.97	Sigma–Aldrich	y = 16,078,791x + 423,632	0.929	4.5–3575.3
Linalool	0.97	Sigma–Aldrich	y = 119,097,534x + 3,404,283	0.990	0.2–802.9
α-Terpineol	0.99	Sigma–Aldrich	y = 117,434,169x + 316,143	0.997	4.5–277.1
Geraniol	0.99	Sigma–Aldrich	y = 154,230,237x + 248,291	0.999	1.1–461.7
Citral	0.97	Sigma–Aldrich	y = 3,558,639x + 991,708	0.900	22.0–857.6
Geranylacetone	0.95	Sigma–Aldrich	y = 31,704,911x−466,013	0.997	23.7–329.6

Supplementary Table S2. Identification of volatile compounds in two pummelo cultivars cultivated in different regions of China.

No.	Volatile Compound	Retention Index	ID	Quantitative Ion (*m/z*)	Quantitative Standard
1	Hexanal	1086	A	56	Hexanal
2	E-2-Pentenal	1137	C	55	2-Hexenal
3	Heptanal	1183	B	56	Hexanal
4	2-Hexenal	1222	A	83	2-Hexenal
5	Z-2-Heptenal	1331	C	41	2-Hexenal
6	Nonanal	1392	A	57	Nonanal
7	E,E-2,4-Hexadienal	1403	B	81	2-Hexenal
8	E-2-Octenal	1433	B	41	2-Hexenal
9	E,E-2,4-Heptadienal	1499	B	81	2-Hexenal
10	Benzaldehyde	1528	A	106	Benzaldehyde
11	E-2-Nonenal	1534	C	43	2-Hexenal
12	Pentanol	1255	A	42	Pentanol

13	Z-2-Penten-1-ol	1324	B	57	Z-3-Hexen-1-ol
14	Hexanol	1347	A	56	Hexanol
15	Z-3-Hexen-1-ol	1384	A	41	Z-3-Hexen-1-ol
16	1-Octen-3-ol	1451	A	57	1-Octen-3-ol
17	1-Hexanol, 2-ethyl	1484	A	57	1-Hexanol, 2-ethyl
18	Octanol	1556	A	56	Octanol
19	Methyl Isobutyl Ketone	1025	C	43	2-Heptanone
20	1-Penten-3-one	1037	C	55	2-Heptanone
21	5-Hepten-2-one, 6-methyl	1339	B	108	2-Heptanone
22	Ethyl Acetate	881	A	43	Ethyl acetate
23	Butyl acetate	1074	A	43	Butyl acetate
24	Ethyl octanoate	1437	A	88	Ethyl octanoate
25	Ethyl decanoate	1640	A	88	Ethyl decanoate
26	Butyl butanoate	1908	C	71	Ethyl decanoate
27	Isobutyl 2,2,4-trimethyl-3-carboxyisopropyl pentanoate	1932	C	71	Ethyl decanoate
28	2-Methyl-, 2,2-dimethyl-1-(2-hydroxy-1-methylethyl)propyl propanoate	1948	C	71	Ethyl decanoate
29	β-Myrcene	1162	A	93	β-Myrcene
30	Limonene	1201	A	68	D-Limonene
31	Terpinolene	1289	A	136	Terpinolene
32	β-Elemen	1643	B	161	Terpinolene

33	(-)-Germacrene D	1728	B	161	Citral
34	α-Muurolene	1731	B	161	α-Ionone
35	Copaene	1732	B	161	α-Ionone
36	δ-Cadinene	1794	B	161	α-Ionone
37	β-Neoclovene	>2400	B	204	α-Ionone
38	cis-Linalool oxide	1447	A	59	cis-Linaloloxide
39	trans-Linalool oxide	1468	B	59	cis-Linaloloxide
40	Linalool	1546	A	93	Linalool
41	Terpinen-4-ol	1603	A	71	Terpinolene
42	α-Terpineol	1703	A	59	α-Terpineol
42	Geraniol	1856	A	69	Geraniol
43	E-Carveol	1869	B	84	α-terpineol
45	Neral	1687	A	69	Citral
46	Citral	1746	A	69	Citral
47	Geranylacetone	1862	A	69	Geranylacetone
48	Toluene	1056	C	105	p-Cymene
49	Benzene, 1-methyl-4-(1-ethylethenyl)	1440	C	104	p-Cymene
